# Remarkably stable amorphous metal oxide grown on Zr-Cu-Be metallic glass

**DOI:** 10.1038/srep18196

**Published:** 2015-12-14

**Authors:** Ka Ram Lim, Chang Eun Kim, Young Su Yun, Won Tae Kim, Aloysius Soon, Do Hyang Kim

**Affiliations:** 1 Metallic Materials Division, Korea Institute of Materials Science, 797 Changwondaero, Seongsan-Gu, Changwon, Gyeongnam 642-831 Korea; 2 Global E3 Institute and Department of Materials Science and Engineering, Yonsei University, 50 Yonsei-Ro, Seodaemun-Gu, Seoul 120-749 Korea; 3 Center for Non-crystalline Materials, Department of Materials Science and Engineering, Yonsei University, 50 Yonsei-Ro, Seodaemun-Gu, Seoul 120-749 Korea; 4 Department of Optical Engineering, Cheongju University, 36 Naedock-Dong, Cheongju 360-764 Korea

## Abstract

In the present study, we investigated the role of an aliovalent dopant upon stabilizing the amorphous oxide film. We added beryllium into the Zr_50_Cu_50_ metallic glass system, and found that the amorphous oxide layer of Be-rich phase can be stabilized even at elevated temperature above *T*_g_ of the glass matrix. The thermal stability of the amorphous oxide layer is substantially enhanced due to Be addition. As confirmed by high-temperature cross-section HR-TEM, fully disordered Be-added amorphous layer is observed, while the rapid crystallization is observed without Be. To understand the role of Be, we employed *ab-initio* molecular dynamics to compare the mobility of ions with/without Be dopant, and propose a disordered model where Be dopant occupies Zr vacancy and induces structural disorder to the amorphous phase. We find that the oxygen mobility is slightly suppressed due to Be dopant, and Be mobility is unexpectedly lower than that of oxygen, which we attribute to the aliovalent nature of Be dopant whose diffusion always accompany multiple counter-diffusion of other ions. Here, we explain the origin of superior thermal stability of amorphous oxide film in terms of enhanced structural disorder and suppressed ionic mobility due to the aliovalent dopant.

So far, amorphous metal oxides have been synthesized mainly in the form of nanoscale powders or thin films^1^,^2^,^3^,^4^, since scale-up is usually very limited due to their high bulk Gibbs free energy compared to crystalline counterparts^5^,^6^,^7^. However, amorphous metal oxides offer distinctive attractions due to their unique properties such as superior dielectric properties and corrosion resistance compared to the crystalline counterparts^8^,^9^,^10^,^11^,^12^, which are caused by the structural and chemical homogeneity of the amorphous structure. For some glassy alloys, it has been reported that thick amorphous metal oxide films (up to several tens of nanometers) can be obtained just by surface oxidation^13^,^14^,^15^,^16^. Recent research demonstrated that the amorphous metal oxide film grows up to thickness of ~8 nm on Ni_62_Nb_38_ glassy alloy surface under carefully controlled annealing condition^17^, by which friction and wear properties of the sample are significantly enhanced. In addition, ~70 nm thick Zr–based amorphous oxide film has been reported in Zr–Cu–Al–Be glassy alloy system^13^, which is a substantial improvement compared to the amorphous phase of pure zirconia film, where the growth of such amorphous layer is limited to only ~1 nm^7^. Still, the role of the minor component in the glassy alloy system has not been investigated in detail yet. Considering such a drastic improvement on the order of magnitude of the growth thickness, it is of greater fundamental interest to investigate the effect of aliovalent dopants.

The aim of the present study is to suggest the key factors for obtaining stable amorphous metal oxide film. In addition to the well-established experimental approaches, we employed computational modeling and quantum mechanical calculations to elucidate the role of the aliovalent dopant in the glass matrix. Zr_50_Cu_50_ binary glassy alloy, which is one of the most widely studied glass forming alloys, was selected for the present purposes. As demonstrated in our previous study^13^, aliovalent dopants significantly enhance the thermal stability of the amorphous oxide layer. In the case of Cu_45_Zr_45_Al_8_Be_2_ glassy alloy, two aliovalent dopants (Al and Be) are co-doped into the amorphous zirconia film. The thermal stability of the amorphous phase can be achieved by destabilizing the crystalline phases. According to the previous report^18^,^19^, the solid solubility in crystalline zirconia is strongly influenced by valences and sizes of the multivalent cations, which means that aliovalent dopants with low solubility in crystalline zirconia can stabilize the amorphous structure by destabilizing the crystalline phases. Between Al and Be, Be dopant is considered to play a key role in stabilizing the amorphous structure of the Zr-rich oxide film, since Be (+2) has much lower solid solubility in crystalline zirconia than Al (+3), considering the number of valence electrons and the ionic sizes. Here we focus on the role of Be dopant, which has a different oxidation state of Be (2+) compared to major glass component Zr (4+), and conduct a series of systematic analyses using both of the experimental and theoretical tools.

The aliovalent dopant is found to form completely different atomic arrangement in the glass network, resulting in substantially enhanced structural disorder. Also, due to the aliovalent nature, diffusion of the dopant requires simultaneous counter-diffusion of multiple ions, which adds another energetic cost to the phase separation. Moreover, the Be ions used in this study are found to suppress the oxygen mobility of the amorphous layer compared to its crystalline counterparts.

## Methodology

To investigate the alloying effect on structural characteristics of the thermally grown oxide film, Zr_50_Cu_50_ and Zr_46_Cu_46_Be_8_ amorphous ribbons about 5 mm wide and 50 μm thick were fabricated by melt-spinning under Ar atmosphere with an operating pressure of 0.05 MPa. The oxidation behavior of these melt-spun ribbons was examined by thermogravimetric analysis (TGA, Perkin Elmer TGA 4000), and the thermal properties of the samples were measured using differential scanning calorimetry (DSC, Perkin Elmer DSC 7) at a heating rate of 40 K/min. Thermal analysis reveals that the glass transition temperatures (*T*_g_) and the crystallization onset temperatures (*T*_x_) are 685 K and 738 K for the Zr_50_Cu_50_ metallic glass, and 675 K and 741 K for the Zr_46_Cu_46_Be_8_ metallic glass, respectively. To investigate the structural characteristics of the initial oxide film, the ribbon samples were continuously heated in TGA furnace (the net flow rate of dry air was kept constant at 20 cm^3^/min during heating) up to the target temperatures at a heating rate of 40 K/min. Detailed microstructural and compositional investigations were carried out using transmission electron microscopy (TEM, JEOL 2010F), equipped with a high angle annular dark field (HAADF) detector and an energy dispersive spectrometer, X-ray diffractometry (XRD, Bruker AXS, D8 DISCOVER), and dynamic secondary ion mass spectrometry (D-SIMS, CAMECA IMS-4FE7). To observe the full cross-section of oxidized surface, the samples were prepared using a focused ion beam milling equipment (Dual-beam FIB, FEI Helios NanoLab).

In order to understand how beryllium stabilizes the amorphous structure in the amorphous zirconia, we employed *ab-initio* molecular dynamics as introduced by Parrinello and Rahman^20^,^21^, where the heat-matter interaction is simulated by the Langevin thermostat^22^. The calculations were carried out within density functional theory (DFT) method in the generalized gradient approximation (GGA) given by Perdew, Berke and Ernzerhof (PBE)^23^,^24^ with projector-augmented-wave (PAW) method^25^ as implemented in VASP code^26^,^27^. The kinetic energy cutoff of 400 eV and Monkhorst-Pack^28^ grid of 2 × 2 × 2 were employed.

The 2 × 2 × 2 supercell of cubic phase zirconia is chosen for the model of the pure zirconia at high temperature. In Be-doped zirconia model, eight Be atoms are introduced to randomly chosen eight Zr-O vacancies sites, forming Be_x_Zr_(1−x)_O_(2−x)_ solid solution model, resulting in 9.1 at% of Be in the glass matrix, corroborating with the sample used in the experimental section (8 at% of Be). Initially, a high control temperature close to melting temperature of pure zirconia was set for 2,000 ionic relaxation steps in order to introduce thermal disorder into the model, then the temperature was brought down to standard temperature for another 2,000 steps, followed by annealing at 773K for additional 4,000 steps. After the simulated-annealing procedures, the final structures of pure and Be-doped zirconia are compared in terms of long-range disorder as described by the radial distribution function (RDF).

The calculation trajectory can be projected into statistical analysis in order to visualize the effect of Be in terms of ionic mobility. The motion of ions throughout the trajectory should be considered as a Markovian process, because the Langevin thermostat relies on random forces to control the temperature, and also because the inertia of particles is not explicitly considered. In a Markovian process the momentum is not conserved, and each snapshot of the trajectory can be sought as an independent statistical sample. Based on this, we projected the velocity trajectories of the simulation onto the temperature-mobility space through Gaussian filter (the broadening parameter along the mobility axis is taken from the Maxwell-Boltzmann distribution, 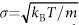, where σ denotes the broadening parameter, κ_B_ the Boltzmann constant, *T* temperature, and *m* the atomic mass of the ions). The resulting probability distributions give qualitative comparison between the mobility of the ions with/without beryllium in the oxide. More quantitative interpretation can be obtained as we further calculate the first moment center of the probability along the mobility axis.

## Results and Discussion

**Figure 1:**
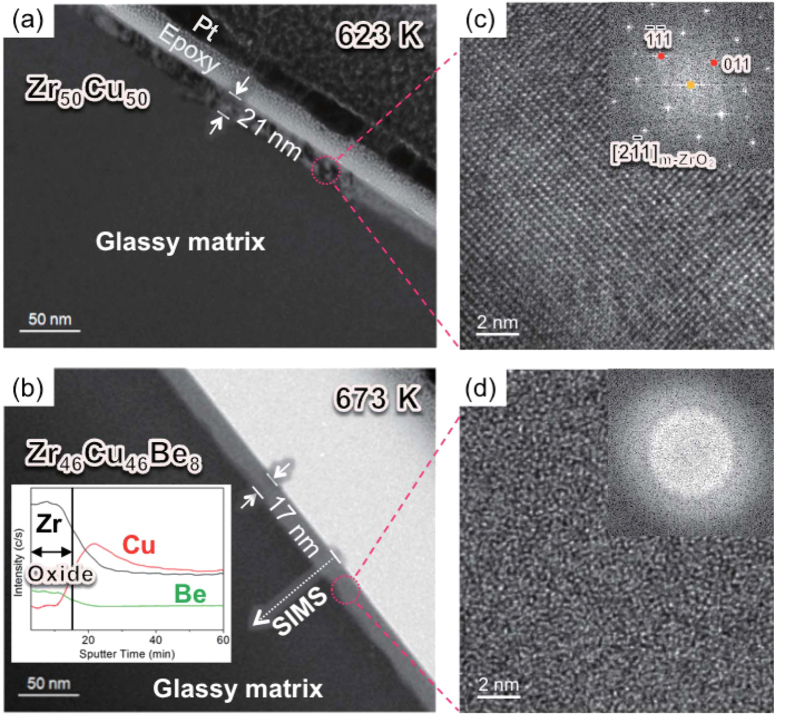
Cross-sectional bright field TEM images obtained from (**a**) the Zr_50_Cu_50_ and (**b**) the Zr_46_Cu_46_Be_8_ alloys showing the oxide films after continuous heating up to 623 K and 673 K, respectively; and the magnified HR TEM images of the oxide film and the FFT diffraction patterns (Fig. 1(c,d)) obtained from the region marked by the dashed line in Fig. 1(a,b).

Figure 1(a,b) show cross-sectional bright-field (BF) TEM images obtained from the Zr_50_Cu_50_ and Zr_46_Cu_46_Be_8_ ribbon samples after continuous heating up to 623 K and 673 K, respectively. Figure 1(c,d) show the high-resolution (HR) TEM images obtained from the regions marked by the dotted line in Fig. 1(a,b), respectively. The HR TEM images and their fast Fourier transformed (FFT) diffraction patterns (insets in Fig. 1(c,d)) reveal that the oxide film of the Zr_50_Cu_50_ alloy has a crystalline structure (monoclinic type) but that of the Zr_46_Cu_46_Be_8_ alloy has an amorphous structure. The result of SIMS depth profiling for the amorphous oxide film indicates that the amorphous oxide consists mainly of Zr and O and contains small amount of Be (see the inset of Fig. 1(b)). As shown in the SIMS depth profile, Cu atoms diffuse and accumulate beneath the amorphous oxide film. Considering the standard Gibbs free energies for formation of oxides (−197.7, −977.7 and −1096.4 kJ/mol of O_2_ for CuO, ZrO_2_ and BeO, respectively, at 623 K and under the standard state pressure of 1 bar^29^), the result of composition analysis can be considered natural, since Be is expected to be most preferentially reacted with oxygen in the system, and therefore easily soluble in the amorphous oxide.

**Figure 2:**
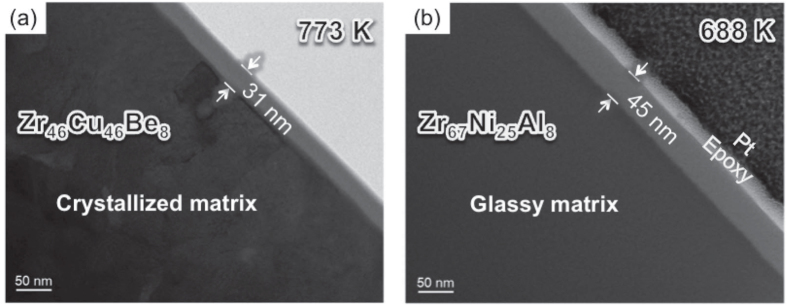
Cross-sectional bright field TEM images obtained from (**a**) the Zr_46_Cu_46_Be_8_ and (**b**) the Zr_67_Ni_25_Al_8_ alloys showing the thickness of the oxide films after continuous heating up to 773 K (32 K above *T*_x_) and 688 K, respectively.

Figure 2(a) shows a cross-sectional BF TEM image obtained from the Zr_46_Cu_46_Be_8_ ribbon sample after continuous heating up to 773 K. In the case of the amorphous oxide film, no contrast is observed because the amorphous oxide contains none of the crystalline defects such as grain boundaries and dislocations. It is interesting that the oxide film still retains amorphous structure even after the matrix is crystallized. Previous research reported that crystallized substrate causes the crystallization of the amorphous oxide film due to the high crystalline–amorphous interface energy compared to the amorphous–amorphous interface energy in Zr-based metallic glasses (such as Zr–Cu–Al and Zr–Ni–Al)^14^. In the case of Zr–Cu–Be metallic glass, however, the crystallized substrate did not induce the crystallization of the amorphous oxide film. Thus, in this case, the crystallization of the amorphous oxide film is considered to be suppressed by kinetic reasons (mainly by low ionic diffusivities) rather than thermodynamic reasons.

Basically, it is known that m-ZrO_2_ and BeO are the most stable phases in the Zr–Be–O system^30^,^31^, so the amorphous oxide certainly is a thermodynamically unstable phase in this system. In this case, however, a phase separation step is essentially required to form the two most stable crystalline phases, since m-ZrO_2_ and BeO are insoluble in each other. Therefore, the amorphous phase can be stable as long as phase separation into Zr-rich and Be-rich phases does not occur. Elemental partitioning for the phase separation requires long-range diffusion of transport ionic species, so it depends on the ionic diffusivities in the system. To examine the dynamic behavior of ionic species in amorphous zirconia, ab initio molecular dynamics calculation was conducted.

**Figure 3:**
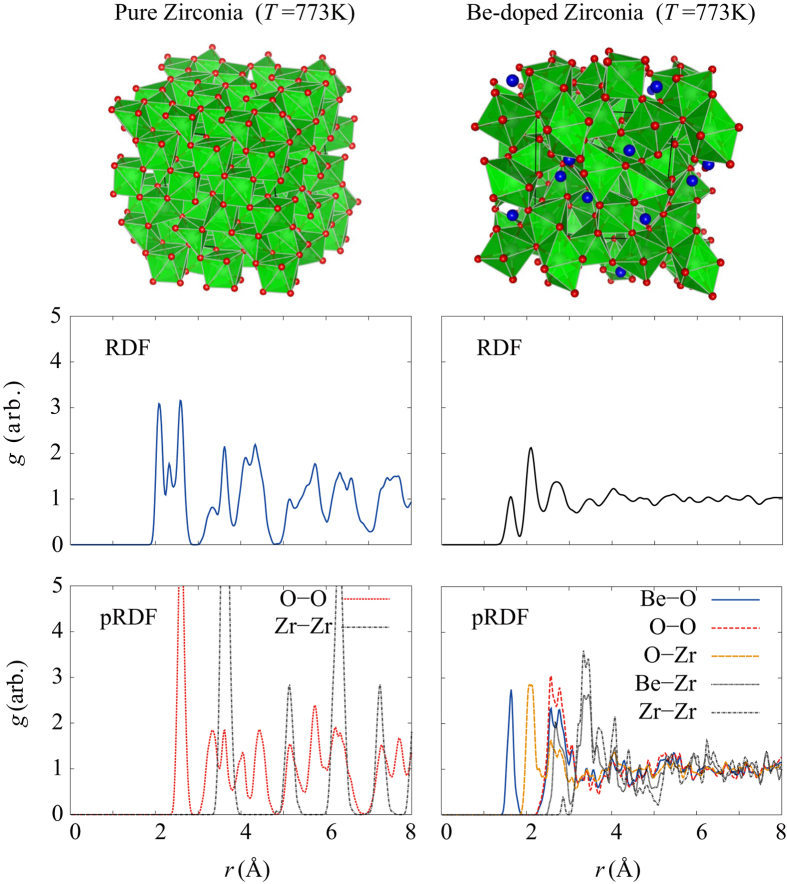
The atomic structure of pure/Be-doped zirconia models at 773 K. The radial distribution functions (RDFs) visualize the short and long-range order of the model structures, emphasizing the role of Be to enhancing the long-range disorder of the structure as depicted in partial RDF (pRDF).

Figure 3 exhibits the atomic structure of pure/Be-doped zirconia models at 773 K (well above the *T*_x_ of the Zr_46_Cu_46_Be_8_ metallic glass). The beryllium atoms occupy the central position of the neighbored oxygen atoms. This contributes to the enhanced long-range disorder in the oxide due to the difference in number of valence electrons between zirconium and beryllium elements. The pure zirconia showed higher regularity as shown in the calculated radial distribution function. On the other hand, the beryllium led to the formation of Be-O bonding, which results in turn strengthened long-range disorder of the oxide.

**Figure 4:**
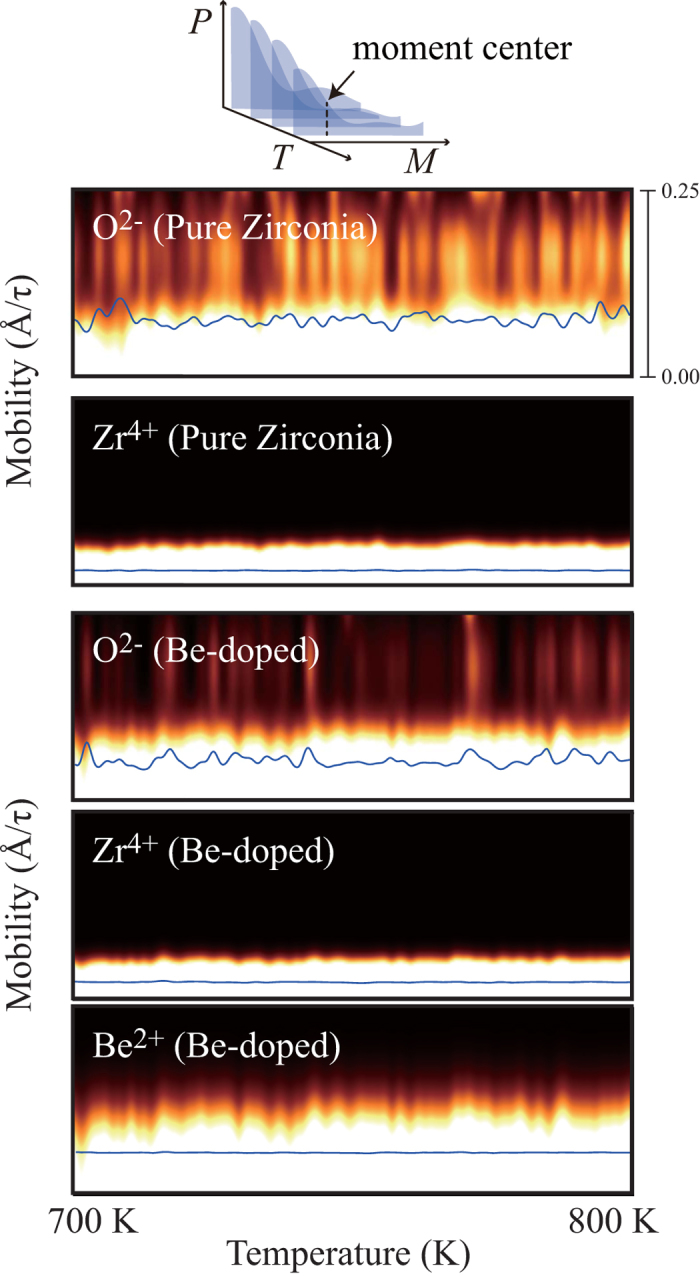
Calculated temperature-mobility distribution from the *NpT* dynamics calculations. Each datum from a snapshot is projected through two dimensional Gaussian filter, where the broadening parameter along the vertical axis is taken from broadening parameter in Maxwell-Boltzmann distribution. The horizontal broadening factor is taken to be 1.0 K. The result demonstrates drastically reduced mobility of oxygen in the case of Be-doped disordered phase. *τ* represents the time step used in the study, 1 *fs*.

In addition to the role of Be in enhancing structural disorder of oxide, beryllium also contribute to suppressing the ionic mobility of oxygen. From the results shown in Fig. 4, the mobility of ions at high temperature (700 ~ 800 K) is expressed by the brightness of the point. The Zr^4+^ ions tend to show much lower mobility compared to O^2−^ and Be^2+^. A clear difference can be found as we compare the mobility of O^2−^ in pure zirconia to those in Be-doped zirconia. The first moment center (line in the figure) shows drastic decrease for the case of Be-doped zirconia. Interestingly, Be^2+^ shows comparable mobility to O^2−^, however, the fluctuation pattern is more similar with that of Zr^4+^. When a Be^2+^ displaces, the change in the local ionic charge should be compensated by diffusion of other cations, most likely Zr^4+^ because the concentration of Be^2+^ is not high in the oxide, and this might suppress the mobility of Be^2+^, even lower than O^2−^ in the pure zirconia model. Likewise, the mobility of Zr^4+^ can be affected by Be^2+^. Hence, once Be^2+^ and Zr^4+^ are mixed into an amorphous state, phase transformation from the amorphous phase to the crystalline phases (ZrO_2_ and BeO) is difficult due to the low mobility of cations. Compared to the Al-doped amorphous zirconia film formed on Zr_67_Ni_25_Al_8_ alloy (see Fig. 2(b)), the Be-doped amorphous zirconia film was found to be relatively thin even at higher temperatures, which can be another evidence to support that Be-doped amorphous zirconia has relatively low ionic diffusivities.

**Figure 5:**
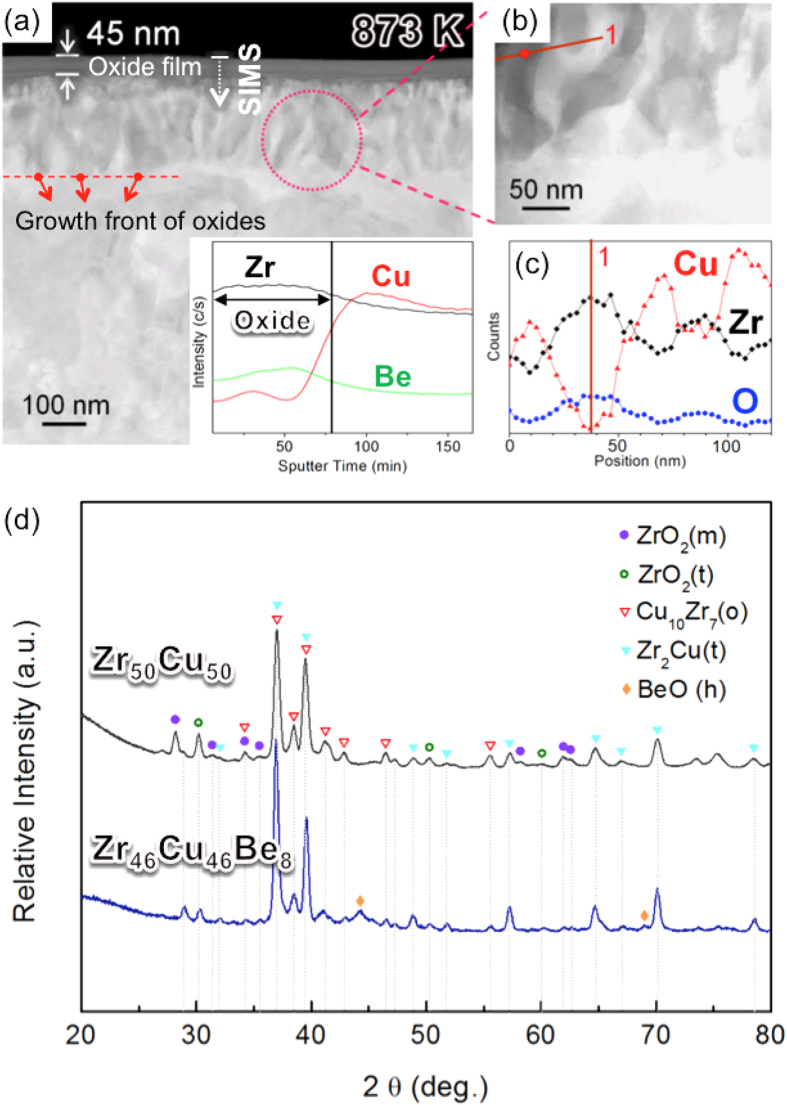
(**a**) HAADF STEM image obtained from the Zr_46_Cu_46_Be_8_ alloy after continuous heating up to 873 K; the inset is a SIMS depth profile obtained from the area marked by the arrow; (**b**) the magnified image obtained from the region marked by the dotted line in Fig. 5(a); (**c**) the EDS line scan profile obtained from the solid line in Fig. 5(b); (**d**) the X-ray diffraction patterns obtained from the Zr_50_Cu_50_ and Zr_46_Cu_46_Be_8_ ribbon samples after continuous heating up to 873 K.

Figure 5(a) shows a HAADF scanning transmission electron microscopy (STEM) image obtained from the Cu_46_Zr_46_Be_8_ sample after continuous heating up to 873 K. Though the thickness of the oxide film increases up to ~45 nm with further oxidation up to 873 K, the oxide films still retains amorphous structure (confirmed by HR TEM images and their fast Fourier transformed diffraction patterns, not shown here). The result of SIMS depth profiling (see the inset of Fig. 5(a)) reconfirms that the amorphous oxide film contains small amount of Be and the growth of the oxide film accompanies with the rejection of Cu. Figure 5(b) shows the magnified image obtained from the region marked by the dotted line in Fig. 5(a), and Fig. 5(c) shows the EDS line scan profile obtained from the solid line in Fig. 5(b). The results of the EDS line scan profile indicate that subsurface region (~230 nm thick) beneath the oxide film divides into the oxides (dark region in Fig. 5(b)) and Cu-enriched phases (bright region in Fig. 5(b)). The X-ray diffraction patterns, obtained from the Zr_50_Cu_50_ and Zr_46_Cu_46_Be_8_ ribbon samples after continuous heating up to 873 K (see Fig. 5(d)), reveal that crystalline oxides (such as monoclinic zirconia, tetragonal zirconia and beryllium oxide) begin to form beneath the oxide film between 773 K and 873 K.

From this, we can obtain two important pieces of information. First, it is considered that the amorphous oxide film can no longer well-protect its substrate from the oxidation around 873 K. From Deal-Grove model^32^, it is expected that the rate determining factor changes from the ionic diffusion through the amorphous oxide film to the back diffusion of Cu at the growth front of oxides when the diffusivities of transported species in the oxides become large relative to the rate constants (such as the gas-phase transport coefficient and the surface-reaction rate constant for oxidation). The growth fronts of oxides shown in Fig. 5(a) imply that the increased oxygen permeability through the oxide film makes the direction of the diffusion field change from inward the oxide film to outward. The change of direction in the diffusion field forms unstable protrusions on the planar interface, which breaks the planar oxidation front into cells. Second, the presence of BeO suggests that if the diffusivities of the constituent elements in the alloy matrix become large enough for elemental partitioning, further oxidation proceeds via the formation of crystalline ZrO_2_ and BeO instead of Be-doped amorphous zirconia.

Generally, the growth of oxide films occurs by chemical reactions at the interfaces (the gas-oxide interface and the oxide-material interface) and transport of mobile elements through the oxide layer. In the case of crystalline zirconia (n-type oxide), it is known that oxidation proceeds via the migration of oxygen ions through the oxide layer, since oxygen ion has much higher diffusivity than Zr^4+^ 33. On the contrary, in the case of amorphous zirconia, outward diffusion of Zr cations becomes important for oxide-film growth, since the movement of oxygen ions is limited due to a very low oxygen ion vacancy concentration^33^,^34^. So, in this case, Zr^4+^ is considered as a transport ionic species. In the case of Be-doped Zr-based amorphous oxide, however, the transport species changes from cations (Zr^4+^ and Be^2+^) to an anion (O^2−^) below 873 K. It is considered that the cation mobility is strongly affected by local ionic structure. Even at elevated temperatures, the movement of cations can be limited by the difference in the valence charge of the ions, since the diffusion of Be^2+^(Zr^4+^) should accompany with simultaneous diffusion of Zr^4+^(Be^2+^). In other words, cations are bound to imaginary static charge.

## Conclusion

We investigated the effect of beryllium addition into the Zr_50_Cu_50_ metallic glass system on the glass stability of thermally grown amorphous oxide film. The results of experiments revealed that both oxidation reaction and crystallization of amorphous oxide film are strongly suppressed by the addition of beryllium to the Zr-Cu alloy. The role of beryllium ions in the amorphous zirconia has also been investigated by *ab-initio* molecular dynamics coupled with NpT langevin thermostat. In the calculated results, Be ions occupy central position of the neighbor oxygen ions, increasing the long-range disorder of the oxide. In this case, the movement of the cations (Be^2+^, Zr^4+^) is very limited to maintain local charge neutrality. It is considered that the low mobility of the cations is a key factor for increasing the glass stability of the amorphous oxide film, which corresponds well to the experiment results.

## Additional Information

**How to cite this article**: Lim, K. R. *et al*. Remarkably stable amorphous metal oxide grown on Zr-Cu-Be metallic glass. *Sci. Rep*. **5**, 18196; doi: 10.1038/srep18196 (2015).
